# Optical coherence tomography for an in-vivo study of posterior-capsule-opacification types and their influence on the total-pulse energy required for Nd:YAG capsulotomy: a case series

**DOI:** 10.1186/1471-2415-14-131

**Published:** 2014-11-18

**Authors:** Gregor Hawlina, Darko Perovšek, Brigita Drnovšek-Olup, Janez Možina, Peter Gregorčič

**Affiliations:** Eye Hospital, University Medical Centre Ljubljana, Grablovičeva 46, 1525 Ljubljana, Slovenia; Faculty of Mechanical Engineering, University of Ljubljana, Aškerčeva 6, 1000 Ljubljana, Slovenia

**Keywords:** Capsular bag distension syndrome, Capsulotomy, High-resolution spectral-domain optical coherence tomography, Nd:YAG, Posterior capsule opacification

## Abstract

**Background:**

Posterior capsule opacification (PCO) is the most common post-operative complication associated with cataract surgery and is mostly treated with Nd:YAG laser capsulotomy. Here, we demonstrate the use of high-resolution spectral-domain optical coherence tomography (OCT) as a technique for PCO analysis. Additionally, we evaluate the influence of PCO types and the distance between the intraocular lens (IOL) and the posterior capsule (PC), i.e., the IOL/PC distance, on the total-pulse energy required for the Nd:YAG laser posterior capsulotomy.

**Methods:**

47 eyes with PCO scheduled for the Nd:YAG procedure were examined and divided into four categories: fibrosis, pearl, mixed type and late-postoperative capsular bag distension syndrome. Using custom-made computer software for OCT image analysis, the IOL/PC distances in two dimensions were measured. The IOL/PC distances were compared with those of a control group of 15 eyes without PCO. The influence of the different PCO types and the IOL/PC distance on the total-pulse energy required for the Nd:YAG procedure was analyzed.

**Results:**

The total-pulse energy required for a laser capsulotomy differs significantly between PCO types (*p* = 0.005, Kruskal-Wallis test). The highest energy was required for the fibrosis PCO type, followed by mixed, pearl and late-postoperative capsular bag distension syndrome. The IOL/PC distance also significantly influenced the total-pulse energy required for laser capsulotomy (*p* = 0.028, linear regression). Lower total-pulse energy was expected for a larger IOL/PC distance.

**Conclusions:**

Our study indicates that the PCO types and the IOL/PC distance influence the total-pulse energy required for Nd:YAG capsulotomy. The presented OCT method has the potential to become an additional tool for PCO characterization. Our results are important for a better understanding of the photodisruptive mechanisms in Nd:YAG capsulotomy.

## Background

Posterior capsule opacification (PCO) is the most common post-operative complication associated with modern cataract surgery [1], with a reported incidence of approximately 28% after 5 years [2]. PCO usually develops due to the epithelial cells of the lens being left behind in the capsular bag after any type of extracapsular cataract surgery [3]. Different experimental studies [4–6] have suggested that the posterior capsule (PC) itself does not opacify. PCO occurs as a result of the formation of opaque secondary membranes by proliferation, migration, epithelial-to-mesenchymal transition, collagen deposition, and lens fiber regeneration of the lens epithelial cells [7]. The lens epithelial cells proliferate in several patterns. Clinically, there are two basic morphological types of PCO, the fibrosis type and the pearl type, which have different cellular origins [3, 8, 9]. The fibrosis-type PCO is caused by the proliferation and migration of the lens epithelial cells, which undergo an epithelial-to-mesenchymal transition, resulting in fibrous metaplasia [8]. In contrast, the pearl-type PCO is formed by the lens epithelial cells located at the equatorial lens region. These cells generate the regeneration of crystallin-expressing lenticular fibers and the formation of Elshing pearls and a Soemmering ring [3, 9].

Another post-operative complication of cataract surgery (phacoemulsification) that also affects the visual function is late-postoperative capsular bag distension syndrome (CBDS). According to Miyake et al. this syndrome is characterized by the accumulation of liquefied material in a closed space between the PC and the intraocular lens (IOL) optic formed due to the occlusion of the anterior capsular opening created during the cataract surgery [10].

The most effective treatment for PCO and late-postoperative CBDS is Nd:YAG laser capsulotomy. The procedure involves clearing the visual axis by creating a central opening in the opacified PC [3, 11]. This procedure involves focusing a Nd:YAG laser pulse, with an energy of several millijoules and a duration of several nanoseconds, immediately behind the PC. Previous studies [12–17] have reported that the side effects are more pronounced when higher single-pulse energy, rather than higher total-pulse energy, is used. To avoid unwanted IOL damage, these studies all proposed that the Nd:YAG laser posterior capsulotomy should be performed with the lowest possible single-pulse energy.

The main focus of our study was to investigate how *(i)* the different PCO types and/or CBDS and *(ii)* the distance between the IOL and the PC (*IOL/PC distance*) affect the total-pulse energy required to create the posterior capsulotomy. For this purpose the analysis of different PCO types was required in relation to the total-pulse energy used for each capsulotomy.

## Methods

### Patients

Our prospective study was performed at the Eye Hospital, University Medical Centre Ljubljana, Slovenia and was approved by the National Ethics Committee. All the patients provided written, informed consent. The study protocol adhered to the tenets of the Declaration of Helsinki.

The present study was performed over 7 months and included patients with biomicroscopically detectable PCOs and/or late-postoperative CBDS who were instructed to have Nd:YAG laser capsulotomy by ophthalmologists. The patients were chosen for treatment if they had reduced visual acuity with capsule opacity, glare or monocular diplopia. All the procedures were performed more than one year after uneventful phacoemulsification cataract surgery, and all the patients had implanted foldable silicone, hydrophobic or hydrophilic acrylic IOLs. Statistical analysis regarding PCO formation and IOL positioning of the different IOL models were not performed. Patients with active ocular disease, a previous Nd:YAG laser posterior capsulotomy, IOL displacement, sulcus-fixated IOL, PC rupture, aphakia, and those who had difficulty with ocular fixation with OCT were excluded from our study.

Forty-seven eyes of 40 patients with PCO were included in the study. The patients ranged in age from 59 to 89 years (mean age: 76.3 years; standard deviation (SD), 7.9); 20 were men (mean age: 77.2 years; SD, 6.7) and 15 were women (mean age: 74.9 years; SD, 9.5).

The control group comprised 15 eyes of 12 patients (8 were men and 4 were women) without PCO on whom uneventful cataract surgery had been performed. The control group (mean age: 76.8 years; SD, 11.8) was analyzed several months after the cataract surgery and was used for a comparison with the IOL/PC distances measured in patients with different PCO types. For a more comprehensive reading we classified late-postoperative CBDS as another type of PCO.

### Patient examination

**Figure 1:**
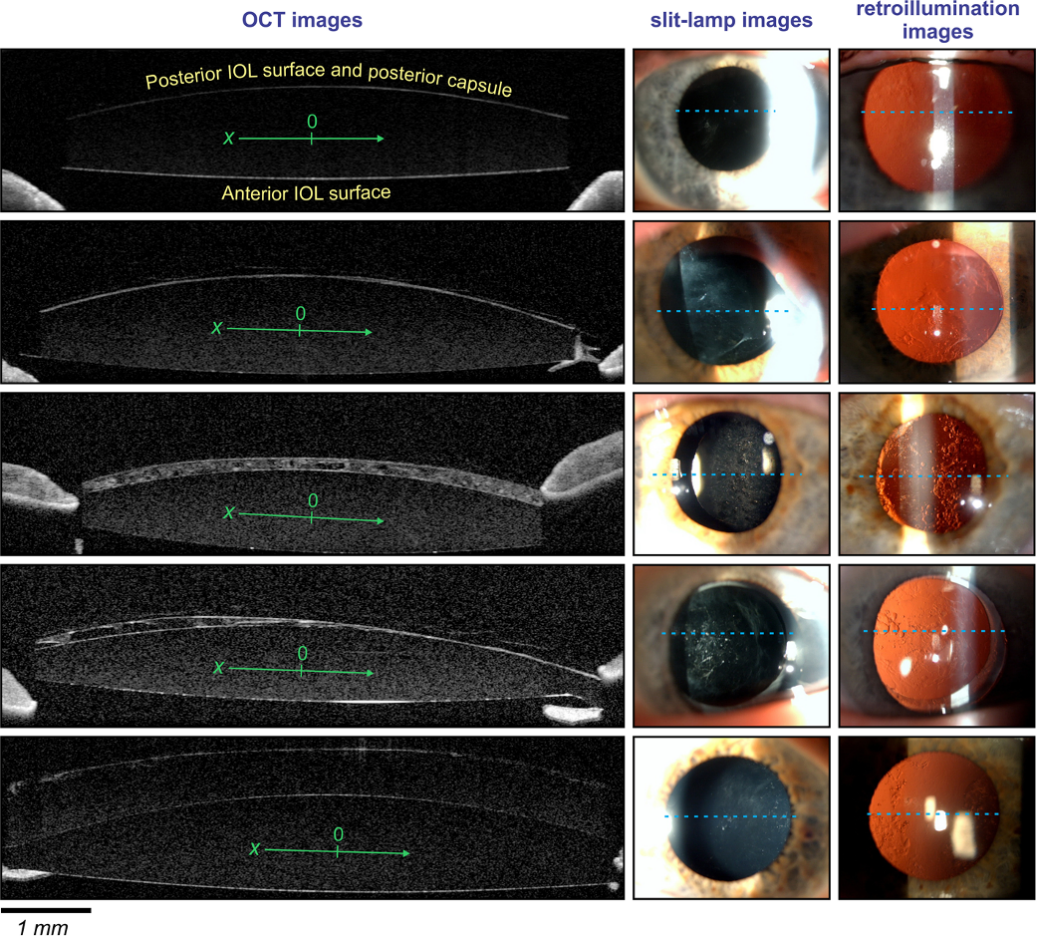
**Typical OCT (first column), slit-lamp (second column) and retroillumination images (third column) of IOL without PCO at 7 months after cataract surgery (first row), and different PCO types: fibrosis-type PCO (second row), pearl-type PCO (third row), mixed-type PCO (fourth row), and late-postoperative CBDS (fifth row).** The dashed lines in the slit-lamp and retroillumination images show the position of the OCT cross-section image. The arrows within the IOL on the OCT images show the horizontal IOL direction, while the zero corresponds to the IOL center.

In all patients, anterior segment biomicroscopy with a PCO evaluation was performed by one ophthalmologist (G.H.) under pharmacological mydriasis. Before the Nd:YAG laser procedure was initiated, an OCT examination (Figure 1, first column), slit-lamp photography (Figure 1, second column), and retroillumination photography (Figure 1, third column) were performed by a certified assistant research technician (D.P.). According to the slit-lamp evaluation and the analysis of the OCT image, the PCO type was defined.Following the slit-lamp evaluation, the PCO was segregated into one of the four groups: fibrosis (Figure 1, second row), pearl (Figure 1, third row), mixed type (Figure 1, fourth row), and late-postoperative CBDS (Figure 1, fifth row).Mixed-type PCO was defined as PCO with the characteristics of both the fibrosis and pearl types, visible under the slit-lamp and on the OCT image. A typical mixed-type PCO is presented in Figure 1 in the fourth row, where the left-hand side of the PCO is recognized as a pearl type, while the right-hand side of the PCO is a fibrosis type.

We segregated some cases, where different amounts of transparent or turbid, milky fluid was collected in the capsular bag into clinical entity late-postoperative CBDS. Late-postoperative CBDS was mostly characterized upon slit-lamp examination by a fibrotic, thickened anterior capsular opening and by the accumulation of a liquefied substance between the IOL and the PC. In some cases, when the distance between the IOL and the PC was not large enough, the CBDS type was recognized during the Nd:YAG capsulotomy procedure. Here, in all of our CBDS cases, a surge of viscous fluid was seen coming out of the gap between the IOL and the PC, leaking into the vitreous. Thus, this leakage was a characteristic finding in all of our CBDS cases.

### OCT examination

OCT examinations were performed before the Nd:YAG laser procedure on a high-resolution spectral-domain OCT (Spectralis HRA + OCT, Heidelberg Engineering Inc., Germany). To perform the OCT analysis on the plane of the PC, we used infrared light (for a background image of the patient’s eye) with an anterior segment lens in 30° settings on a high-resolution line scan and in the angle modality. Mostly high-resolution line scans were stretched across 30° covering 16.7 mm; otherwise, three-dimensional scans of 11 lines covering 11.1 mm on 30° were used. For the analysis, the horizontal OCT line scan through the center of IOL optic was used. The center of the IOL optic was determined by using the OCT line scan with the highest distance between anterior and posterior IOL surface. Typical OCT images are shown in the first column of Figure 1, where the line scan position is indicated in the second and the third column with a dashed line.

### Nd:YAG-Laser capsulotomy procedure

All the procedures were performed by one ophthalmologist (G.H.). For this purpose, we employed a Q-switched Nd:YAG laser with a 7.6 ns pulse duration (Modified Optimis laser system made by Optotek d.o.o., Ljubljana, Slovenia).

**Figure 2:**
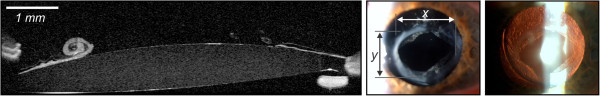
**An OCT (left), slit-lamp (middle) and retroillumination image (right) captured immediately after the laser capsulotomy of a mixed-type PCO.** This case before the procedure is presented in Figure 1 (fourth row). The slit-lamp image (middle) also serves as a schematic demonstration of the *x* and *y* axes of the “rhombus” that outlines the area of the posterior capsule opening.

In all cases, we induced mydriasis and used a 150-μm backfocus. However, as a precaution, the aiming beam was first focused slightly away from the PC, toward the retina. If there was no visible damage on the PC at the starting focus position, the aiming beam was carefully moved backwards, i.e., nearer to the PC, until visible damage was seen. In all of our patients, we employed a capsulotomy contact glass (CGPL lens, Haag Streit) and used a cruciate pattern to perform the capsulotomy. We aimed the size of the capsulotomy to be greater than 4.0 mm in the horizontal and vertical direction (see Figure 2, middle image). According to the previous studies and recommendations [12–17] to use rather lower single-pulse energy even in return of higher total-pulse energy, we used the single-pulse energy just above the threshold for optical breakdown of our laser system. All the procedures were successfully finished with the same single-pulse energy, *E*_*p*_ = 1.6 mJ.

Many Nd:YAG laser capsulotomy techniques have been described in the literature and used in clinical practice. Our study takes into account a survey of UK practices and recommendations made by Gomaa and Liu [18].

### Total-pulse energy measurements

While performing the Nd:YAG laser procedure in each eye, we counted the number of laser pulses, *N*_*p*_, that induced an optical breakdown (i.e., the plasma spark was visible). The total-pulse energy, *E*_*tot*_, required to create the PC opening was defined as *E*_*tot*_ = *N*_*P*_*E*_*P*_. Here, *E*_*p*_ stands for the single-pulse energy.

After the procedure, the size of the capsulotomy was measured in *x*-*y* coordinates under a slit-lamp with accuracy better than 200 μm (e.g., see Figure 2, middle image). In this way we obtained the length of the *x* and *y* diagonals of a rhombus outlining the PC opening and calculated the area of the capsulotomy as rhombus area 

For a more objective comparison of the results obtained in different eyes, we normalized the total-pulse energy, *E*_*tot*_, by the measured area of the capsulotomy *A*. Thus, for each procedure, we calculated the total-pulse energy per area 

### Analysis of the IOL/PC distance

**Figure 3:**
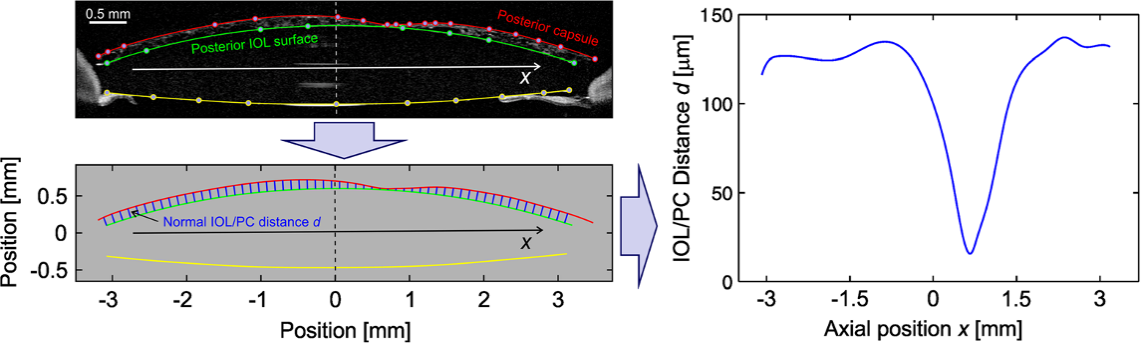
**Schematic presentation of the OCT image processing protocol to obtain the IOL/PC distance as a function of the axial lens position** ***x*** **.** The shape of the PC and the posterior IOL surface is first approximated by a Bézier spline, a series of cubic Bézier curves joined end-to-end in control points. The control points (the white points in the top left picture) are manually selected so that the Bézier spline (the red and green curves in the top left picture) coincides with the shape of the PC and the posterior IOL surface. The Bézier spline is formed from equidistant points. For each point, the normal distance between the IOL and PC is calculated, as is shown schematically (only for selected points) with the blue lines in the bottom left picture. The calculated IOL/PC distance distribution as a function of the axial position *x* is shown in the right picture.

OCT images were first exported from our database using Eye Explorer (Heidelberg computer program for viewing OCT images) and were then processed to obtain the IOL/PC distance distribution in two dimensions. Here, the resolution of our method was limited by the size of one pixel of the exported image, which is 4 μm in the vertical and 11 μm in the horizontal direction of the OCT image. Custom-made software, written in MATLAB, was designed (P.G.) for the purpose of image processing. We developed and used this software for semi-automatic measurements of the IOL/PC distance distributions. The main principle of our image processing is schematically represented in Figure 3. Here, the shape of the PC and the posterior IOL surface was first approximated by a Bézier spline, [19] i.e., a series of cubic Bézier curves joined end-to-end in control points. The control points (the white points in the top left picture in Figure 3) were manually selected by an ophthalmologist (G.H.) so that the Bézier spline (the red and green curves in the top left picture in Figure 3) coincided with the shape of the PC and the posterior IOL surface.

**Figure 4:**
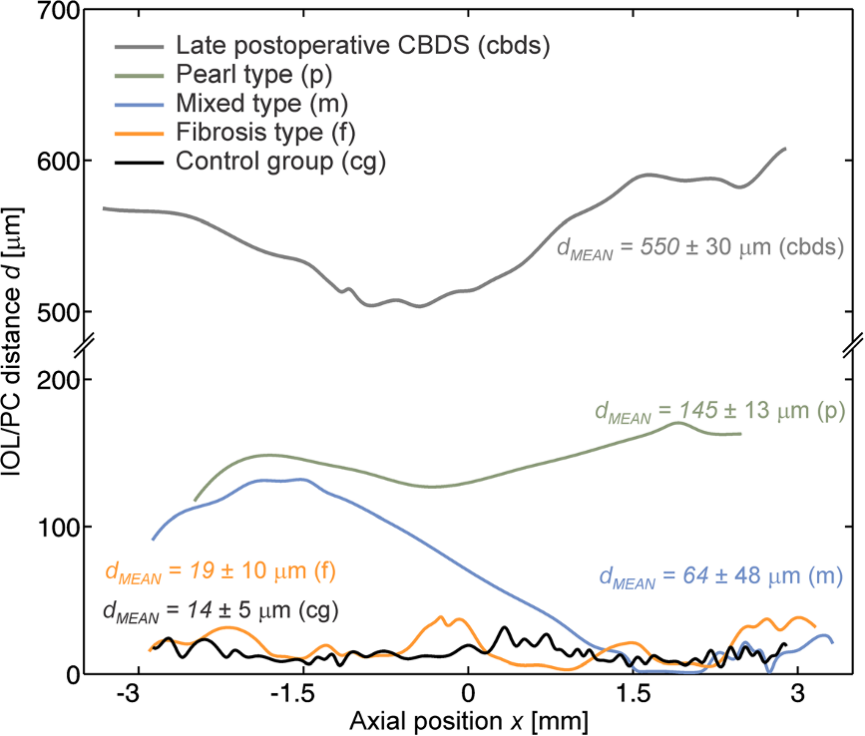
**IOL/PC distance distribution as a function of the axial lens position** ***x,*** **calculated from the typical OCT images in Figure** 1 **, i.e., for different PCO types: late-postoperative CBDS, the pearl type, mixed type, and fibrosis type, as well as for the IOL without PCO at 7 months after the cataract surgery (the black curve).**

The Bézier spline is formed by equidistant points. For each point, the normal distance between the IOL and PC was calculated, as is shown schematically (only for selected points) with the blue lines in the bottom-left picture in Figure 3. The calculated IOL/PC distance distribution is shown as a function of the axial position *x* in the right-hand picture in Figure 3.The IOL/PC distance distributions for different PCO types, calculated from the typical OCT images in Figure 1, are presented in Figure 4.

### Data analysis

To compare the IOL/PC distances between different PCO types, we calculated (for each OCT image) a mean IOL/PC distance *d*_*MEAN*_ from the measured two-dimensional IOL/PC distance distribution. Henceforth, we will call this *mean* IOL/PC distance (obtained from a single OCT image) simply the IOL/PC distance.

For each PCO type, we calculated the median, minimum and maximum of both the IOL/PC distances (obtained from a single OCT image) and the total-pulse energy per area. These values are listed in Tables 1 and 2. Because the distributions were not normal, we used the Kruskal-Wallis test for statistical analysis of the differences in the IOL/PC distances between PCO types. This test was also used to compare the total-pulse energy per area that was needed to create a posterior capsulotomy in different PCO types. The comparison of the IOL/PC distance between the fibrosis-type PCO and the control group was performed using the Mann–Whitney test.

**Table 1:** **IOL/PC distances for different PCO types**

	IOL/PC distance [μm]		
PCO type	***Median***	***Minimum***	***Maximum***
Control group	11.5	8.5	14.8
Fibrosis	25.4	8.8	49.4
Pearl	112.1	54.7	236.0
Mixed	83.1	40.3	146.8
Late-postoperative CBDS	104.6	29.9	543.7

The values obtained for the control group are also listed in the first row. All the listed values are in units of μm.

**Table 2:** **Total pulse-energy per area for different PCO types**

	Total-pulse energy per area [mJ/mm ^2^ ]		
PCO type	***Median***	***Minimum***	***Maximum***
Fibrosis	14.20	5.5	34.5
Pearl	5.75	3.8	12.9
Mixed	8.35	4.5	17.7
Late-postoperative CBDS	5.85	2.4	7.2

All the listed values are in units of mJ/mm^2^.

A linear regression was used to analyze the influence of the IOL/PC distance on the total-pulse energy per area.

In our study, we considered that *p* values below 0.05 were statistically significant. We performed the statistical analysis using the R statistical package (version 2.15).

## Results

A group of 47 eyes with PCO and 15 eyes without PCO were analyzed: 11 cases (23%) were segregated into fibrosis-type PCOs, 16 cases (34%) into pearl-type PCOs, 12 cases (26%) into mixed-type PCOs, and 8 cases (17%) into the late-postoperative CBDS. The fifteen cases without PCOs served as a control group.

**Figure 5:**
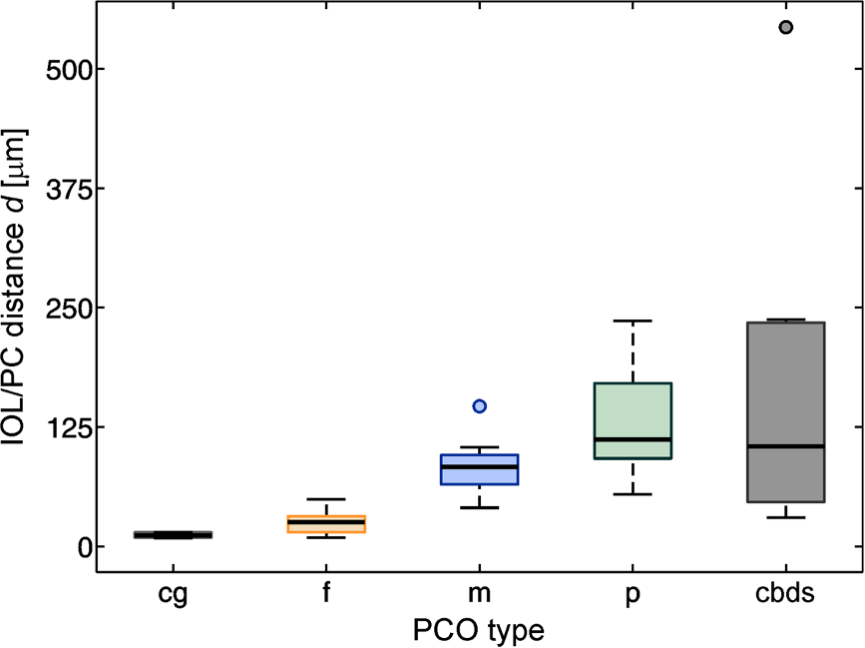
**The distribution of the IOL/PC distances for the control group (cg) and for the different PCO types: fibrosis (f), mixed (m), pearl (p) and late-postoperative CBDS (cbds).** The differences between the groups are highly significant (*p* <0.001).

The IOL/PC distances for each PCO type were collected and analyzed. The statistical data for the IOL/PC distances are listed in Table 1. The distributions of the IOL/PC distances are presented in the box plot in Figure 5. The differences in the IOL/PC distances between the different PCO types are statistically highly significant (*p* <0.001).The results in Figure 5 show that the pearl type (p) had the highest median IOL/PC distance, followed by the late-postoperative CBDS (cbds), the mixed (m), the fibrosis type (f), and the control group (cg).

**Figure 6:**
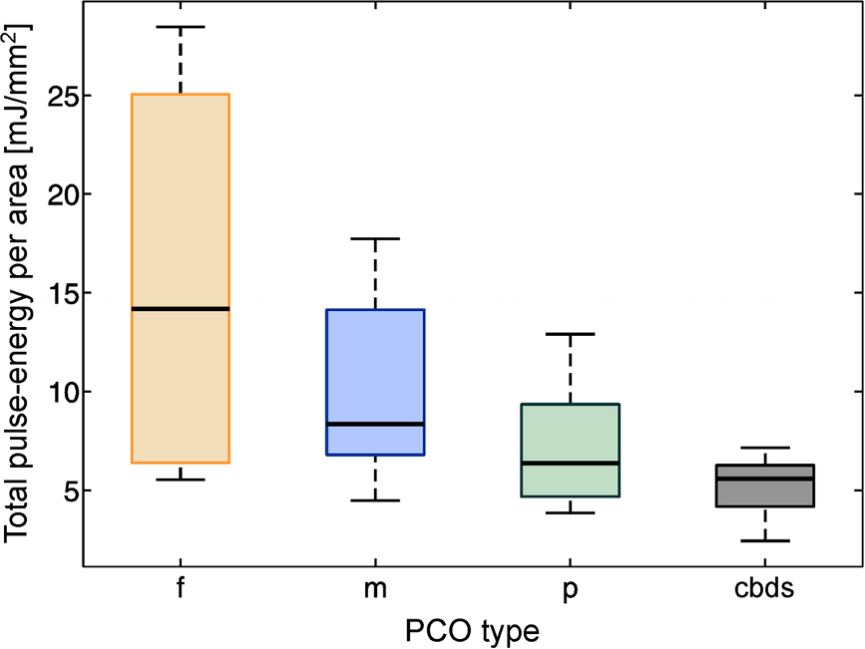
**The distribution of the total-pulse energy per area for different PCO types: fibrosis (f), mixed (m), pearl (p) and late-postoperative CBDS (cbds).** The groups differ significantly (*p* = 0.005).

Figure 6 shows the box plot of the total-pulse energy per unit area that was needed to perform a posterior capsulotomy for different PCO types. This distribution differs significantly between PCO types (*p* = 0.005). The statistical data for this comparison are listed in Table 2. The highest median total-pulse energy per area is required to treat the fibrosis PCO type, followed by the mixed, the pearl and the late-postoperative CBDS.

**Figure 7:**
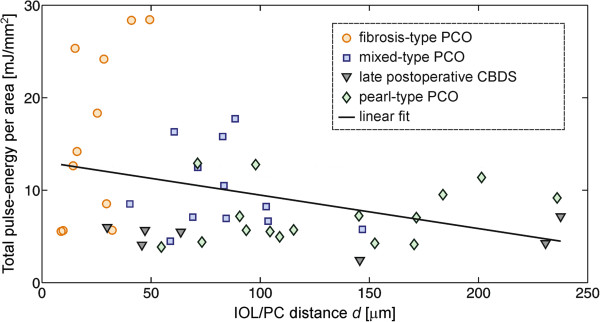
**The total-pulse energy per area as a function of the IOL/PC distance. Different PCO types are represented by different marks: the orange circles show the fibrosis type; the blue squares are the mixed type; the gray triangles correspond to the late-postoperative CBDS; and the green rhombuses correspond to the pearl-type PCO.** The black line shows a linear fit. The results are statistically significant (*p* = 0.028).

The total-pulse energy per area as a function of the IOL/PC distance is presented in Figure 7. The solid line shows a linear fit to all the plotted data, where we excluded one case of the late-postoperative CBDS whose IOL/PC distance equaled 544 μm. This case was recognized as an outlier (cbds type in Figure 6). Thus, the inference from our results can only be made for patients with IOL/PC distances in the range between 9 μm and 238 μm. The estimated regression coefficient equals −36 mJ/mm^3^ (*p* = 0.028) and the 95% confidence interval for the regression coefficient equals [−68, 5] mJ/mm^3^. Therefore, this result is also statistically significant.

## Discussion

In our study, we showed *(i)* the use of high-resolution spectral-domain OCT for PCO characterization, *(ii)* the OCT characteristics of different PCO types, and *(iii)* the influence of PCO types and the IOL/PC distance on the total-pulse energy required to create posterior capsulotomy using an Nd:YAG laser.

### High-resolution spectral-domain OCT for PCO characterization

We used high-resolution spectral-domain OCT images to show the characteristics of different PCO types (e.g., see Figure 1). OCT facilitates the high-resolution cross-sectional imaging of the tissue [20, 21] and was thus used for additional PCO characterization. With our OCT method, we were able to distinguish different types of PCO and to measure the IOL/PC distance in two dimensions. Until now ultrasound biomicroscopy (UBM) [22–24] and Scheimpflug imaging [25, 26] have mostly been used for the evaluation of PCO, late-postoperative CBDS and IOL positioning in the capsular bag. OCT has rarely been used to analyze a PC or PCO [20, 27–29]. These few studies mostly employed OCT with a lower resolution. In this context, we present high-resolution spectral-domain OCT as a tool for future studies of changes to the PC following an Nd:YAG laser capsulotomy procedure.

It has been demonstrated previously [30, 31] that OCT has sufficient reproducibility and can also be used to analyze anterior segment features [20, 32–34], including the PC [20, 27–29, 35, 36]. Moreno-Montañes et al. [29] presented OCT with a lower resolution as a method for the objective evaluation of PCO. Using OCT, these authors estimated the PCO intensity and thickness in patients after cataract surgery. Although they showed good and acceptable interoperator repeatability for measuring posterior capsule thickness, there is still no commercially available application for PCO characterization using OCT. To obtain the IOL/PC distance map, automatic software would substantially help to reduce the amount of time needed to obtain the type of results presented in our study. Proper software would be useful for a three-dimensional analysis of different PCO types, PCO development in different IOLs, and studies of the IOL position after cataract surgery. Because the OCT has no observer bias [29] and allows a more objective analysis than slit-lamp images, the OCT method also has the potential to become an additional tool in grading systems for PCO. However, for these purposes, automatic image processing is required to speed up the image analysis and to allow the measurement of the IOL/PC distance over the selected area (not only a single-line scan).

### OCT characteristics of different PCO types

The literature refers mainly to two basic PCO types: the fibrosis and pearl types. These two basic morphological types are characterized by cell types that are responsible for their development [37]. The results of our OCT measurements of IOL/PC distances show that the distance distribution in these two basic PCO types is practically uniform, i.e., it is practically independent of the axial position.In clinical practice, in some cases, the slit-lamp examination shows the characteristics of both basic PCO types. For this reason, we included mixed-type PCO, as a type where pearl-type and fibrosis-type PCOs are found within the same PCO. The mixed-type PCO can be easily recognized from the OCT images by the irregular IOL/PC distance (Figure 1, fourth row). In this case, the distance distribution (the third graph from the top in Figure 4) varies from the values that are typical for the pearl type (the left-hand side of the graph) to the values that are typical for the fibrosis type (the right-hand side of the graph).

During our procedures, we observed in some patients a gap between the IOL and the PC that was filled with a transparent or milky fluid. These cases correspond to late-postoperative CBDS, described by Miyake et al. [10]. The amount of fluid was different for the various cases: in some cases, the accumulated fluid pushed the PC posteriorly and the gap was obvious; in other cases, the eyes disclosed only a small amount of fluid [38]. Using a slit-lamp, most of these cases combined the morphological characteristics of a fibrosis-type PCO and late-postoperative CBDS. When the IOL/PC distance was large enough, a gap between the IOL and the PC was visible under the slit-lamp. Otherwise, the gap became more obvious during the procedure, when a surge of fluid flowed into the vitreous. The gap was also clearly visible on the OCT images, which were sometimes required for the analysis before the procedure to find differences with the “ordinary” fibrosis-type PCO. Our observations match cases described by Miyake et al. [38], where late-postoperative CBDS is shown in conjunction with other types of PCO. Because we expect that the IOL/PC gap influences the total-pulse energy required for the Nd:YAG capsulotomy, we recommend using OCT imaging in clinical studies as an additional tool for PCO characterization.

Our OCT method can be used to distinguish late-postoperative CBDS from other types of PCO. While late-postoperative CBDS is a clinical diagnosis, OCT imaging could be useful for precisely documenting the presence of the condition. Our observations are in agreement with those of Tan et al. [39], also based on analysis of case series, where OCT is recommended in the diagnosis of late-postoperative CBDS when the turbid, whitish fluid and the distance between the IOL and PC are not obvious.

To characterize the PCO types based on the IOL/PC distances, we also added a control group composed of patients without PCO after an uneventful cataract surgery. In this case, the PC is in close contact with the IOL, and the median IOL/PC distance is 11.5 μm (minimum 8.5 μm, maximum 14.8 μm) (see Table 1). This value can serve as an estimate of the thickness of the PC after the cataract surgery. Additionally, we compared the IOL/PC distances of the fibrosis-type PCO with the IOL/PC distances measured in the control group (cg and f in Figure 5, and the data listed in Table 1). The difference was statistically significant (*p* = 0.02). However, in the borderline cases, the fibrosis-type PCO could not be recognized only from the OCT image, and a slit-lamp examination was still required.The most common PCO types, i.e., the fibrosis and pearl types, differ not only in the IOL/PC distance but also in how they behave during the Nd:YAG laser procedure. The difference is visible in Figure 2, which shows the OCT, slit-lamp and retroillumination images immediately after the Nd:YAG capsulotomy of the mixed-type PCO. The OCT image in Figure 2 (left) reveals the difference of the PC opening for different PCO types: on the left-hand side a pearl-type PCO is predominant, while the fibrosis type exists on the right-hand side of the PC. The pearl-type PCO mostly retracts and rolls under the IOL, while the fibrosis-type PCO, which consists of myofibroblast-like cells, appears more rigid. Thus, it does not allow the PC to retract away from the laser-induced breakdown site; instead, when treating with laser pulses, the PC acts more like a door on a hinge.

Our results reveal that the IOL/PC distances differ significantly between PCO types. This finding is in agreement with the results presented by Moreno-Montañes et al. [29].

### Influence of the PCO Type and IOL/PC distance on the total-pulse energy

The main goal of our study was to find the influence of *(i)* the PCO types and *(ii)* the IOL/PC distance measured by the OCT system on the total-pulse energy required to create a posterior capsulotomy of a certain area. The relationship between the PCO types and the total-pulse energy per area is presented in Figure 6, and it is statistically significant. Moreover, from the result presented in Figure 7, it can be concluded that the IOL/PC distance also significantly affects the total-pulse energy needed for the capsulotomy. Here, the estimated regression coefficient implies that for a 100-μm increase in the IOL/PC distance, we can expect a decrease of 3.6 mJ/mm^2^ of the total-pulse energy per area. However, the fit of the line is not perfect; in particular, some cases with small distances require a large amount of energy. The small sample size (47 eyes of 40 patients) does not allow us to find a more complex model.

Results of our study indicate that the distance between the PC and IOL plays an important role in the usage of the total-pulse energy during Nd:YAG capsulotomy. We believe that lower total-pulse energy at larger distances is the result of the dynamics of the secondary phenomena (a cavitation bubble and a shockwave) of laser-induced breakdown near different surfaces. Previous *in-vitro* studies [40–42] observed the cavitation bubble and the shockwave near elastic membrane or solid surface. However, according to the results presented in this paper, it would be worth observing these phenomena also in an *in-vitro* experiment where the elastic membrane will be moved some distance away from the solid surface. Thus, the presented results should help to build an *in-vitro* experimental setup, which will be able to provide new insights into photodisruptive mechanisms responsible for the PC cut at different distances from the IOL. Such *in-vitro* results could be clinically applicable for designing the laser system with optimal backfocus for different types of PCO.

### Limitations of the study

IOL material and design play an important role in the development of PCO, as was shown in a number of previous studies [28, 43–46]. Although we documented the IOL model implanted in our patients and took care that the control group had a similar distribution of IOL models to those of the other groups, the groups of patients with the same IOL model were not large enough to provide any statistically significant conclusions about how and if the material and design of the IOL affect the use of energy during Nd:YAG capsulotomy. However, we believe that the influence of IOL material and design on the total-pulse energy required for Nd:YAG capsulotomy deserves a detailed statistical analysis of larger groups of patients with the same IOL model in the future studies.

The horizontal (*x*) and vertical (*y*) dimensions of capsulotomies were measured using the slit-lamp light beam. We approximated the capsulotomy area with rhombus having the diagonals in *x* and *y* axes. However, in the further studies we suggest to calculate this area more accurately by image processing, e.g., as was performed in the study of Kanellopoulos et al. [47].

We also could not create a non-masking design of the study. This limitation could introduce bias from the operator towards the certain type of PCO and the total energy used. To minimize operator bias and to ensure a non-biased study, we strictly defined the study protocol as described in the Methods section.

Due to the similar dependence of the total-pulse energy on *(i)* the PCO type and *(ii)* the IOL/PC distance, we cannot claim that the IOL/PC distance is the only factor affecting the total-pulse energy that is needed to perform a capsulotomy. During our procedures we also found that the repeatability of the laser focus position (with respect to the PC capsule) depends on the patient’s cooperation, the surgeon’s skill, the angle of the capsulotomy contact glass and the quality of the laser system. The influence of the aforementioned working conditions cannot be neglected. For these reasons, we believe that it is worth performing an *in-vitro* experiment, under more reproducible conditions, that takes into account the findings of the present study. We believe that such an *in-vitro* experiment will clarify the isolated influence of the IOL/PC distance on the total-pulse energy and may reveal the main mechanisms that are responsible for the PC opening.

## Conclusions

In the presented study, we used the high-resolution spectral-domain OCT for PCO characterization. Using this technique, we evaluated the influence of the PCO types and the IOL/PC distances on the total-pulse energy required for Nd:YAG capsulotomy.

Our results show a significant influence of *(i)* the PCO types and *(ii)* the IOL/PC distance on the total-pulse energy that is needed to create a posterior capsulotomy of a certain size. The results also indicate that the OCT can be used in clinical studies as an additional tool for PCO characterization. Therefore, the results presented here are important for *(i)* new insights into different PCO types, *(ii)* future investigations of PCO using OCT, and *(iii)* a better understanding of the photodisruptive mechanisms leading to the PC cut at different distances from the IOL.

## Abbreviations

PCO: Posterior capsule opacification
PC: Posterior capsule
CBDS: Capsular bag distension syndrome
IOL: Intraocular lens
Nd: YAG: Neodymium-doped yttrium aluminum garnet
OCT: Optical coherence tomography.
